# Echo and BNP serial assessment in ambulatory heart failure care: Data on loop diuretic use and renal function

**DOI:** 10.1016/j.dib.2016.11.009

**Published:** 2016-11-09

**Authors:** Frank Lloyd Dini, Anca Simioniuc, Erberto Carluccio, Stefano Ghio, Andrea Rossi, Paolo Biagioli, Gianpaolo Reboldi, Gian Giacomo Galeotti, Fei Lu, Cornelia Zara, Gillian Whalley, Pier Luigi Temporelli

**Affiliations:** a Cardiovascular and Thoracic Department, University of Pisa, Pisa, Italy; b Divisions of Cardiology, University of Perugia, School of Medicine, Perugia, Italy; c Cardiovascular and Thoracic Department, Fondazione IRCCS, Policlinico San Matteo, Pavia, Italy; d Department of Biomedical and Surgical Sciences, Cardiology Section, University of Verona, Verona, Italy; e Department of Internal Medicine, University of Perugia, Perugia, Italy; f Institute of Diagnostic Ultrasound, Australasian Sonographers Association, Auckland, New Zealand; g Division of Cardiology, Fondazione Salvatore Maugeri, IRCCS, Veruno, Italy

**Keywords:** Heart failure, Echocardiography, Natriuretic peptides, Loop diuretics, Renal function

## Abstract

We compared the follow-up data on loop diuretic use and renal function, as assessed by serum creatinine levels, and the estimated glomerular filtration rate (eGFR), of two groups of consecutive ambulatory HF patients: 1) the clinically-guided group, in which management was clinically driven based on the institutional protocol of the HF Unit of the Cardiovascular and Thoracic Department of Pisa (standard of care) and 2) the echo and B-type natriuretic peptide (BNP) guided group (patients conforming to the protocol of the Network Labs Ultrasound (NEBULA) in HF Study Group: Pisa, Perugia, Pavia; Verona, Auckland, and Veruno), in which therapy was delivered according to the serial assessment of BNP and echocardiography. Patients whose follow-up was based on standard of care had a significant higher prevalence of worsening renal function, that was likely related to higher diuretic dosages, whilst, a better management of renal function was observed in the echo-BNP-guided group. The data is related to “Echo and natriuretic peptide guided therapy improves outcome and reduces worsening renal function in systolic heart failure: An observational study of 1137 outpatients” (A. Simioniuc, E. Carluccio, S. Ghio, A. Rossi, P. Biagioli, G. Reboldi, G.G. Galeotti, F. Lu, C. Zara, G. Whalley, P.G. Temporelli, F.L. Dini, 2016; K.J. Harjai, H.K. Dinshaw, E. Nunez, M. Shah, H. Thompson, T. Turgut, H.O. Ventura, 1999; A. Ahmed, A. Husain, T.E. Love, G. Gambassi, L.J. Dell׳Italia, G.S. Francis, M. Gheorghiade, R.M. Allman, S. Meleth, R.C. Bourge, 2006) [1], [2], [3].

**Specifications Table**

**Table:** 

Subject area	*Medicine*
More specific subject area	*Cardiology*
Type of data	*Tables*
How data was acquired	*Echocardiography, biochemical testing of serum BNP and creatinine*
Data format	*Analysed*
Experimental factors	*Consecutive patients with chronic heart failure with reduced ejection fraction (EF)*
Experimental features	*Retrospective, multicentre, observational study*
Data source location	*Pisa, Perugia, Pavia; Verona, Auckland, Veruno*
Data accessibility	*Data is with this article*

**Value of the data**

• Echo and BNP guided HF care can provide a tailored approach to the diuretic treatment [1].
• This strategy can prevent worsening renal function in chronic HF and, thus, may impact patients’ survival [2], [3].

## 1 Data

Transthoracic two-dimensional and Doppler echocardiographic examination was carried out with commercial equipments with 2nd-harmonic imaging. BNP concentrations were processed by Alere Triage BNP Test for Beckman Coulter Immunoassay Systems (Alere San Diego Inc., San Diego, CA, USA), a two-site immunoenzymatic (sandwich) quantitative assay. The eGFR was calculated from the simplified formula derived from the Modification of Diet in Renal Disease study. Supplementary Table 1 shows the characteristics of the study groups. Supplementary Table 2 describes the data on diuretic use (furosemide dose) and the changes in renal function [1].

## 2 Experimental design, materials and methods

This was a retrospective, multicenter, observational study that involved 1137 consecutive outpatients (total cohort). In a group of 570 patients with HF (mean ejection fraction [EF]=30%), management was guided according to the presence of echo signs of elevated left ventricular filling pressure and serum BNP levels, while in the other (*n*=567, mean EF=33%), management was based on clinical judgment. In the latter group, echocardiography was repeated only in the case of changes in clinical status.

## Footnotes

Transparency document Transparency data associated with this article can be found in the online version at http://dx.doi.org/10.1016/j.dib.2016.11.009.
