# Supplementary material for: Echo and BNP serial assessment in ambulatory heart failure care: Data on loop diuretic use and renal function
Source: Data Brief. 2016 Nov 9;9:1074–6. doi: 10.1016/j.dib.2016.11.009 (PMC5126128; doi:10.1016/j.dib.2016.11.009)
Supplement: Supplementary file 1 — Supplementary material [file mmc1.doc]

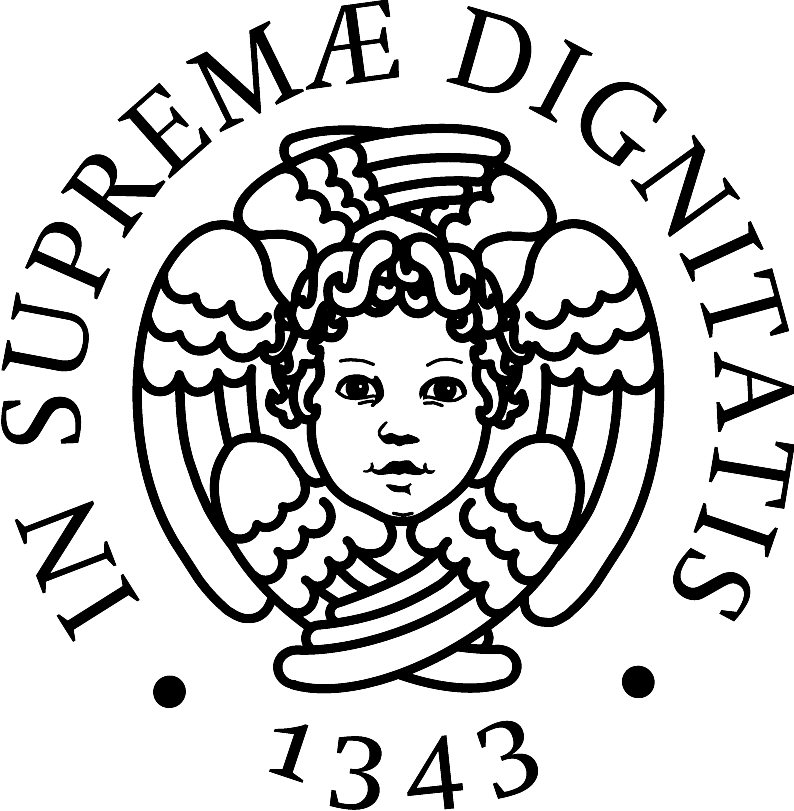


**AZIENDA OSPEDALIERA UNIVERSITARIA PISANA**

**Dipartimento Cardiovascolare e Toracico**

**U.O. di MALATTIE CARDIOVASCOLARI 1a UNIVERSITARIA**

Responsabile: Dott. Frank L. Dini

Tel. 050-99.53.39 - 050-99.53.21 - 329-41.52.340; fax 050-99.53.06


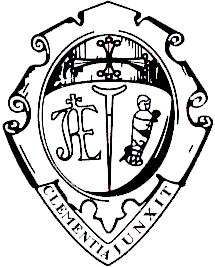


October 7^th^ 2016

Editor Dr. Jiayi

Data in Brief

Data article

**Echo and BNP serial assessment in ambulatory heart failure care: data on loop diuretic use and renal function**

Frank Lloyd Dini^a^, MD Anca Simioniuc^a^, MD, PhD, Erberto Carluccio^b^, MD, Stefano Ghio^c^, MD, Andrea Rossi^d^, MD, Paolo Biagioli^b^, MD, Gianpaolo Reboldi^e^, MD, Gian Giacomo Galeotti^a^, MD, Fei Lu^a^, MD, Cornelia Zara^a^, MD, Gillian Whalley^f^, PhD, Pier Luigi Temporelli^g^, MD, on behalf of the investigators of the Network Labs Ultrasound (NEBULA) in Heart Failure Study Group

**Affiliations:** ^a^Cardiovascular and Thoracic Department, University of Pisa, Pisa, Italy; ^b^Divisions of Cardiology, University of Perugia, School of Medicine, Perugia, Italy; ^c^Cardiovascular and Thoracic Department, Fondazione IRCCS, Policlinico San Matteo, Pavia, Italy; ^d^Department of Biomedical and Surgical Sciences, Cardiology Section, University of Verona, Verona, Italy; ^e^Department of Internal Medicine, University of Perugia, Perugia, Italy; ^f^Institute of Diagnostic Ultrasound, Australasian Sonographers Association, Auckland, New Zealand; ^g^Division of Cardiology, Fondazione Salvatore Maugeri, IRCCS, Veruno, Italy

Herewith, I declare that all the authors have read and approved the manuscript. No authors had conflict of interest regarding this article.

Frank L. Dini, M.D., F.E.S.C.

Please, send further correspondence to:

Dr. Frank L. Dini

Unità Operativa Malattie Cardiovascolari 1

Dipartimento Cardio-toracico

Ospedale Cisanello, Azienda Universitaria-Ospedaliera Pisana

Via Paradisa, 2. 56124 Pisa, Italy

Phone: 39 (050) 995307 and Fax: 39 (050) 995308

E-mail: [f.dini@ao-pisa.toscana.it](mailto:f.dini@ao-pisa.toscana.it)
