# Supplementary material for: Echo and BNP serial assessment in ambulatory heart failure care: Data on loop diuretic use and renal function
Source: Data Brief. 2016 Nov 9;9:1074–6. doi: 10.1016/j.dib.2016.11.009 (PMC5126128; doi:10.1016/j.dib.2016.11.009)
Supplement: Supplementary file 2 — Supplementary material [file mmc2.zip › Supplementary table 1.doc]

| **Table 1 – Comparison between the institutional follow-up protocol of the Heart Failure Unit of the Cardiovascular and Thoracic Department and the protocol of the investigators of the Network Labs Ultrasound (NEBULA) in Heart Failure Study Group** | | |
| --- | --- | --- |
|  | Clinically-guided group | Echo and BNP guided group |
| Clinical History | yes | yes |
| Physical examination | yes | yes |
| Assessment of co-morbid conditions | yes | yes |
| Follow-up BNP assessment | no | yes |
| Follow-up echocardiography | in case of changes in clinical status | if BNP >125 pg/ml |
| Titration of diuretics | empiric | based on echo and BNP levels |

Legend: BNP: B-type natriuretic peptide.
