# Supplementary material for: Echo and BNP serial assessment in ambulatory heart failure care: Data on loop diuretic use and renal function
Source: Data Brief. 2016 Nov 9;9:1074–6. doi: 10.1016/j.dib.2016.11.009 (PMC5126128; doi:10.1016/j.dib.2016.11.009)
Supplement: Supplementary file 2 — Supplementary material [file mmc2.zip › Supplementary table 2.doc]

| **Table 2 – Changes in diuretic dose (furosemide) between baseline and follow-up and the effects on renal function** | | | |
| --- | --- | --- | --- |
|  | **Clinically-guided group** | **Echo and BNP guided group** | **P value** |
| **Number of patients** | 567 | 570 |  |
| **Follow-up duration (days)** | 1274 (IQR: 637-2319) | 1165 (IQR: 513-2076) | NS |
| **Furosemide dose at baseline (mg/day)** | 21 (IQR:11-50) | 25 (IQR:13-50) | NS |
| **Furosemide dose at follow-up (mg/day)** | 36 (IQR:11-86) | 25 (IQR:13-75) | P<0.0001 |
| **Dose increased** | 53% (299) | 26% (145) | P<0.0001 |
| **Dose unchanged** | 28% (159) | 53% (303) | P<0.0001 |
| **Dose decreased** | 19% (109) | 21% (122) | NS |
| **≥ 0.3 mg/dL in serum creatinine** | 22% (127) | 12% (70) | P<0.0001 |
| **Newly diagnosed renal dysfunction (eGFR <60 ml/1.73 m^2^)** | 14% (82) | 12% (70) | NS |

Legend: BNP: B-type natriuretic peptide, eGFR: estimated glomerular filtration rate, IQR: interquartile range, NS: not significant.
